# Clinical Impact of External Carotid Artery Remodeling Following Carotid Artery Stenting

**DOI:** 10.3390/jcm14186682

**Published:** 2025-09-22

**Authors:** Dorota Łyko-Morawska, Michał Serafin, Julia Szostek, Magdalena Mąka, Iga Kania, Wacław Kuczmik

**Affiliations:** Department of General Surgery, Vascular Surgery, Angiology and Phlebology, Faculty of Medical Sciences in Katowice, Medical University of Silesia, 45-47 Ziołowa Street, 40-635 Katowice, Poland; michal.j.serafin@gmail.com (M.S.); jszostek0@gmail.com (J.S.); s81151@365.sum.edu.pl (M.M.); igafaronn@gmail.com (I.K.); wkuczmik@interia.pl (W.K.)

**Keywords:** carotid artery stenting, external carotid artery, cerebral perfusion, collateral circulation, stroke prevention, vascular remodeling, hemodynamics

## Abstract

**Background**: Carotid artery stenting (CAS) is a common revascularization approach for carotid artery stenosis. While its impact on the internal carotid artery (ICA) has been extensively studied, the effects on the external carotid artery (ECA)—a key collateral pathway for cerebral perfusion—remain insufficiently explored. This study aimed to assess structural changes in the ECA following CAS and their clinical significance. **Methods**: A retrospective observational cohort study of 963 patients treated with CAS between 2018 and 2024 was conducted. Demographic data, comorbidities, and procedural characteristics were collected. Pre- and postprocedural ICA and ECA diameters were measured via angiography. Spearman’s correlation, regression modeling, and receiver operating curver (ROC) analysis were used to identify predictors of ECA narrowing and occlusion and their relationship with neurological outcomes. **Results**: The median ECA diameter decreased post-CAS (from 4.7 mm to 3.8 mm, *p* < 0.001). ECA overstenting occurred in 96.4% of cases, with 71.7% exhibiting diameter reduction. De novo ECA occlusion occurred in 2.5% of patients and was associated with a higher incidence of stroke, transient ischemic attack, and in-stent restenosis (ISR). Multivariate analysis identified preoperative ECA diameter (*p* < 0.001), ICA diameter (*p* = 0.001), and second-generation stents (*p* = 0.02) as independent predictors of ECA narrowing. ROC analysis confirmed that a preoperative ECA diameter ≤ 3.05 mm strongly predicted occlusion (Area under the curve (AUC) = 0.93, *p* < 0.001). **Conclusions**: CAS frequently leads to ECA remodeling, including occlusion, compromising collateral perfusion and contributing to adverse ischemic incidences and ISR. Preprocedural ECA assessment may aid in optimizing patient selection and procedural planning.

## 1. Introduction

Carotid artery stenosis remains a major risk factor for ischemic stroke, necessitating effective revascularization strategies to restore cerebral perfusion and reduce long-term morbidity [1,2]. While carotid artery stenting (CAS) primarily targets the internal carotid artery (ICA), its effect on the external carotid artery (ECA) has drawn growing attention [3,4]. The ECA plays a significant role in cerebral hemodynamics through extracranial–intracranial collateral circulation, particularly in patients with advanced ICA disease [5,6,7]. However, post-stenting alterations in ECA dimensions and function remain an underexplored domain [8].

Recent studies suggest that CAS frequently results in partial or complete ECA overstenting, potentially leading to progressive ECA narrowing or occlusion [8]. These vascular changes may have critical implications for collateral perfusion, especially in cases of ICA restenosis or occlusion [9]. Despite this, the clinical impact of ECA remodeling post-CAS, its predictive factors, and its association with long-term stroke risk remain incompletely understood [10].

Therefore, the present study aimed to evaluate structural changes in the external carotid artery (ECA) following carotid artery stenting (CAS) and to identify predictors of post-stenting narrowing or occlusion. Furthermore, we sought to explore the potential clinical implications of these findings in the context of patient outcomes after CAS. By addressing these aspects, our study provides new insights into the anatomical fate of the ECA and its relevance for procedural planning and risk stratification.

## 2. Materials and Methods

### 2.1. Study Characteristics

This retrospective study evaluated patients (*n* = 1132) diagnosed with carotid artery stenosis who were admitted to the Department of General Surgery, Vascular Surgery, Angiology, and Phlebology at the Medical University of Silesia in Katowice, Poland, between February 2018 and December 2024.

The inclusion criteria encompassed patients with carotid artery stenosis confirmed by preoperative Duplex Doppler ultrasonography who underwent primary treatment with CAS. The exclusion criteria comprised patients with carotid artery near-occlusion, unsuccessful CAS (defined as failure to achieve stent deployment due to technical or anatomical factors), those treated with carotid endarterectomy (CEA), and those managed for in-stent restenosis. After applying the exclusion criteria, the analyzed cohort consisted of 963 patients. The decision to perform CAS over CEA was made by the vascular surgery team based on anatomical considerations (e.g., high carotid bifurcation or significant comorbidities), patient risk profile, and institutional preference. No formalized selection algorithm was applied.

### 2.2. Equipment

Carotid artery angiography was performed using the Artis Zee ceiling system (Siemens Healthineers AG, Forchheim, Germany). During CAS procedures, both first-generation (The PRECISE PRO RX^®^, Cordis—single-layer, closed-cell nitinol stent) and second-generation (The CGuard™, InspireMD—dual-layer stent with open-cell nitinol frame and external MicroNet^®^ mesh) carotid stents were utilized. Stent type, length, and diameter were chosen by the lead vascular surgeon based on the specific clinical requirements of each case.

### 2.3. Analyzed Data

Patient characteristics were obtained from the department’s electronic medical record (EMR) system. The following variables were analyzed: demographic data, comorbidities, clinical symptoms, procedural details, and imaging measurements.

Demographic data included age (continuous) and gender (male/female). Comorbidities were assessed based on medical history and diagnosis codes recorded in the EMR. These were defined as follows: arterial hypertension (documented diagnosis or current use of antihypertensives), dyslipidemia (abnormal lipid levels or use of lipid-lowering therapy), and diabetes mellitus (diagnosed type 1 or 2 diabetes or use of antidiabetic medications). Comorbidities were binary (present/absent).

Clinical symptoms were categorized into the following binary variables: history of ischemic stroke, dizziness, headache, transient ischemic attack (TIA), syncope, and tinnitus. Symptomatic ICA stenosis was defined, according to the European Society for Vascular Surgery guidelines, as a history of ischemic stroke or TIA within six months before admission.

Procedural data included the type, diameter, and length of the stent used, as recorded in operative reports. ECA diameter was measured using preoperative and postoperative angiograms. Angiographic measurements were performed in RadiAnt DICOM Viewer (Medixant, Poznań, Poland). The software automatically calibrated vessel diameters using pixel spacing metadata embedded in the DICOM images, ensuring standardized measurements in millimeters. Measurements were taken at the narrowest point between the ECA orifice and the origin of its first branch. All measurements were performed by a single-blinded investigator (Supplementary Materials S1. Protocol for Measurement of ECA and ICA Diameters in Supplementary Materials). ECA overstenting was defined as coverage of the ECA orifice by the carotid stent.

The follow-up period was defined as the time from the CAS procedure to the most recent hospital clinic visit. Follow-up visits were scheduled at 2 weeks, 3 months, 6 months, 1 year, and annually thereafter. Stroke and TIA events during follow-up were identified through EMR review and confirmed by neurologist documentation or neuroimaging consistent with acute cerebral ischemia. In-stent restenosis (ISR) was diagnosed based on Duplex Doppler ultrasound findings indicating > 50% luminal narrowing.

### 2.4. Statistical Analysis

Statistical analysis was performed using Stata^®^ Statistical Software: Release 18 (StataCorp LLC, College Station, TX, USA). The analysis was structured in accordance with the study’s objectives and followed a sequential approach consistent with the order in which the results are presented.

Descriptive statistics were used to summarize baseline characteristics. Qualitative variables were presented as counts and percentages, while quantitative variables were reported as means ± standard deviations (SD) or medians with interquartile ranges (IQR), depending on the data distribution. The Shapiro–Wilk test was used to assess normality.

To compare pre- and post-CAS ECA diameters, the Wilcoxon signed-rank test was applied due to the non-normal distribution of paired data. Spearman’s rank correlation analysis was used to assess associations between ECA diameter changes and continuous or ordinal variables, without assuming linearity.

Based on the correlation results and clinical relevance, selected variables were entered into univariate linear regression models to identify potential predictors of ECA diameter reduction. Variables with *p* < 0.10 in the univariate analysis were included in the multivariate linear regression to determine independent predictors and control for confounding.

Predictors of ECA de novo occlusion were evaluated using univariate logistic regression, followed by multivariate logistic regression including variables significant in the univariate model. Odds ratios (ORs) with 95% Confidence Intervals (CIs) were calculated. For significant quantitative predictors, ROC curves were constructed, and optimal cut-off points were determined using Youden’s index.

Group comparisons (e.g., patients with vs. without ECA occlusion) were performed using Chi-square or Fisher’s exact tests. A *p*-value below 0.05 was considered indicative of statistical significance for all analyses, unless otherwise specified.

## 3. Results

### 3.1. Patients’ Demographics and General Characteristics

The median patient age was 70 years. Comorbidities were observed in 940 (97.6%) patients, with arterial hypertension being the most common (899, 93.4%). Symptomatic stenosis of ICA was observed in 372 (38.6%) patients (Table 1).

### 3.2. Periprocedural Characteristics

The preoperative ICA diameter was 2.8 IQR 1.6 mm and increased to 6.6 mm postoperatively. The most common stents used were second-generation stents (559; 58.1%).

**Figure 1:**
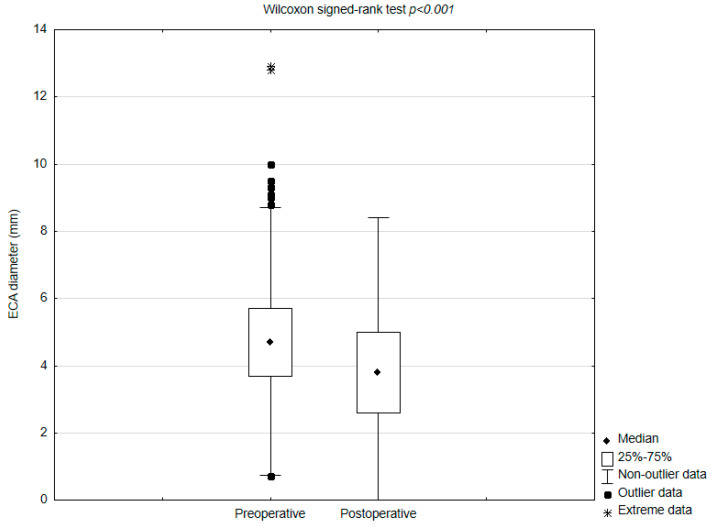
Comparison of pre− and postoperative external carotid artery (ECA) diameters. **Abbreviations:** ECA—external carotid artery.

ECA overstenting occurred in 96.4% of patients. The median ECA diameter decreased from 4.7 IQR 3.8 mm to 3.8 IQR 2.4 mm postoperatively (*p* < 0.001) (Figure 1). A decreased ECA diameter was observed in 666 (71.7%) patients, including 23 (2.5%) with de novo ECA occlusion (Table 2).

### 3.3. Correlations

Spearman’s analysis revealed weak correlations between preoperative ECA and ICA diameter (r = 0.1, *p* = 0.003), preoperative ECA and postoperative ICA diameter (r = 0.12, *p* < 0.001), preoperative ICA and postoperative ECA diameter (r = 0.13, *p* < 0.001), postoperative ECA and ICA diameter (r = 0.17, *p* < 0.001), the difference between pre- and postoperative ECA diameter and preoperative ICA diameter (r = (−0.07), *p* = 0.04), the difference between pre- and postoperative ECA diameter and postoperative ECA diameter (r = (−0.07), *p* = 0.03), and the difference between preoperative ECA diameter pre- and postoperative ECA diameter (r = 0.22, *p* < 0.001). A moderate correlation was observed between the difference between pre- and postoperative ECA diameter and postoperative ECA diameter (r = (−0.44), *p* < 0.001) (Table 3). Although the observed correlations reached statistical significance, they may be partly attributable to the large sample size of our cohort and therefore should be interpreted with caution.

### 3.4. Predictive Factors for ECA Narrowing/Stenosis

In the univariate linear regression analysis for predictive factors for changes in postoperative ECA diameter, the predilatation (R^2^ = 0.004, *p* = 0.04), second-generation stents (R^2^ = 0.006, *p* = 0.02), preoperative ICA diameter (R^2^ = 0.005, *p* = 0.03), and preoperative ECA diameter (R^2^ = 0.097, *p* < 0.001) were statistically significant factors. Therefore they were used in the multivariate analysis. In the multivariate analysis the second-generation stents (*p* = 0.02), preoperative ICA diameter (*p* = 0.001), and preoperative ECA diameter (*p* < 0.001) proved to be independent predictive factors for postoperative changes in ECA diameter (Table 4).

The independent predictive factors found in the multivariate logistic regression analysis were used again in the multivariate logistic regression analysis to prepare the predictive model of changes in postoperative ECA diameter. In the calculated model with a second-generation stent (b = 0.17), preoperative ICA diameter (b = (−0.11)) and preoperative ECA diameter (b = 0.23) were used. The intercept term of the model was −0.08, while R^2^ was equal to 0.11 and *p* < 0.001. The mathematical presentation of the model is presented below.

Change in ECA diameter = 0.17 × (2nd-generation stent = 1, 1st-generation stent = 0) + (−0.11) × preoperative ICA diameter in mm + 0.23 × preoperative ECA diameter − 0.08.

### 3.5. Predictive Factors for ECA Occlusion

In the univariate logistic regression analysis, the stent width (Odds ratio (OR) = 0.56, 95% Confidence Interval (CI) = 0.32–0.98, *p* = 0.04) and preoperative ECA diameter (OR = 0.24, 95% CI = 0.16–0.36, *p* < 0.001) proved to influence the occurrence of ECA occlusion (Table 5).

**Figure 2:**
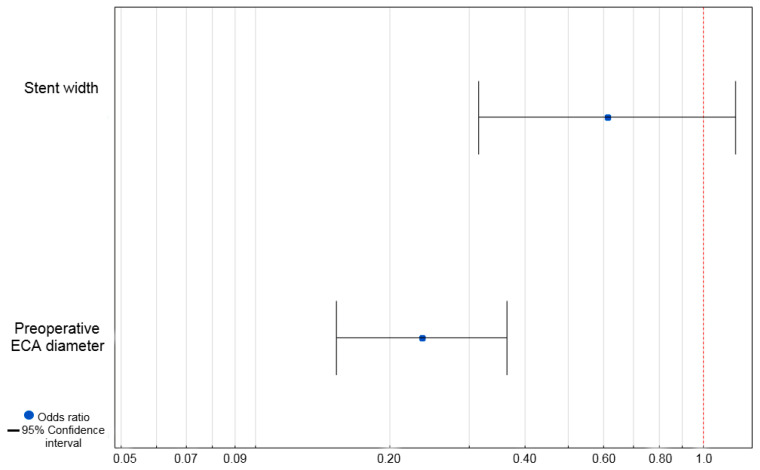
Multivariate logistic regression analysis of predictive factors for ECA occlusion. **Abbreviations**: ECA—external carotid artery.

The statistically significant variables in the univariate analysis were used in the multivariate analysis. In the multivariate analysis only the preoperative ECA diameter proved to be a negative independent predictive factor for ECA occlusion (OR = 0.24, 95% CI = 0.15–0.36, *p* < 0.001) (Figure 2).

**Figure 3:**
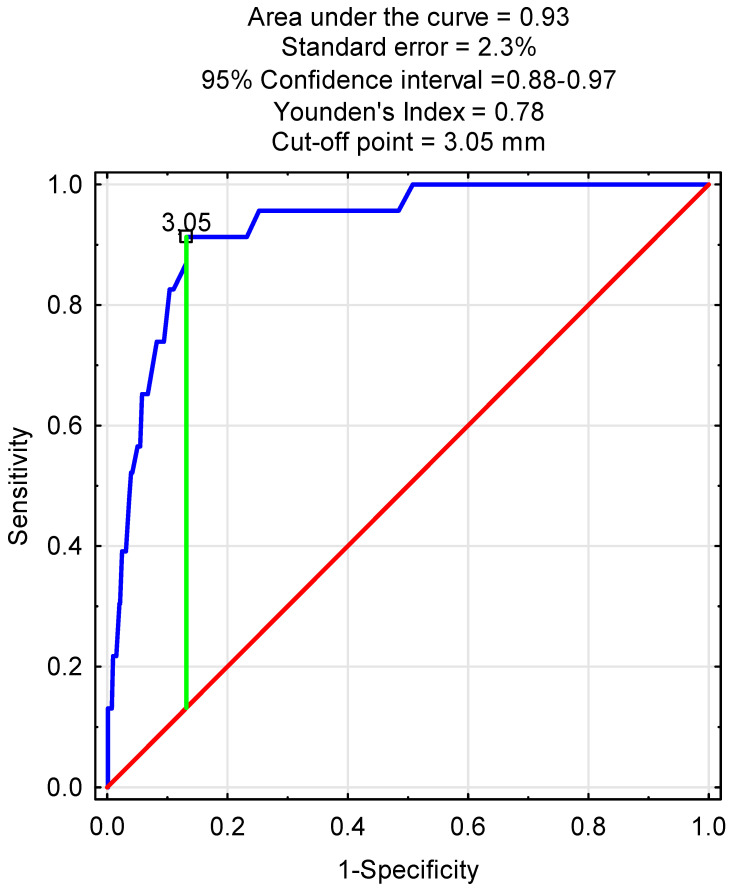
Receiver operating characteristic curve with preoperative ECA diameter for predictions of ECA occlusion (*p* < 0.001).

In the next step the receiver operating characteristic (ROC) curve was calculated with statistically significant quantitative variables in the multivariate logistic regression analysis. In the ROC analysis with preoperative ECA diameter, the area under the curve was equal to 0.93 with a standard error of 2.3%, and the cut-off point was 3.05 mm with *p* < 0.001 (Figure 3).

In the next step the comparative analysis regarding clinical outcomes between patient groups with de novo ECA occlusion (n = 23) and without (n = 940) was performed. Patients with ECA occlusion had higher ipsilateral stroke (2; 8.7% vs. 20; 2.1%, *p* = 0.04), TIA (2; 8.7% vs. 17; 1.8%, *p* = 0.01), and in-stent restenosis (4; 17.4% vs. 23; 2.5%, *p* < 0.001) ratios compared to the patients without ECA occlusion.

There was no statistical significant difference between groups in terms of myocardial infraction (1; 4.4% vs. 27; 2.9%, *p* = 0.68) and mortality (1; 4.4% vs. 13; 1.4%, *p* = 0.24) (Table 6).

## 4. Discussion

In this study, ECA diameter was significantly affected by CAS. ECA overstenting occurred in 96.4% of patients. Additionally, the ECA diameter significantly decreased from 4.7 mm to 3.8 mm postoperatively, with 71.7% of patients showing a reduction in vessel diameter. Interestingly, 2.5% of patients experienced ECA occlusion after the procedure. The use of second-generation stents, a smaller preoperative ICA diameter, and a larger preoperative ECA diameter were identified as significant positive predictive factors for postoperative ECA narrowing. Notably, a larger preoperative ECA diameter (especially above 3.05 mm) was found to be a negative predictive factor for ECA occlusion, with an OR of 0.24. Additionally, patients with ECA occlusion had higher stroke, TIA, and in-stent restenosis rates than patients without ECA occlusion.

The ECA is widely recognized as a crucial source of ipsilateral cerebral blood flow, particularly in the presence of ICA stenosis or occlusion, underscoring its essential role in cerebral perfusion [5,6,7,11]. Previous research has demonstrated that the ECA can supply up to 15% of the total blood flow to the middle cerebral artery [12,13]. Furthermore, a study by Xu et al. [14] showed that performing CAS on the ECA in cases of total ICA occlusion with ipsilateral ECA stenosis can enhance collateral blood flow to the brain, leading to stabilization or improvement of the patient’s neurological status. Therefore, the importance of the ECA should not be underestimated, particularly in the context of ICA stenosis or occlusion.

ECA overstenting was observed in 96.4% of our CAS cases, reflecting a comparable trend of high prevalence, though higher in magnitude, to the 75% reported by De Brost et al. [11]. This reinforces the relevance of ECA monitoring in the CAS setting.

ECA overstenting in 71.7% of our patients resulted in reduced ECA diameter postoperatively. Predictive factors for ECA narrowing included second-generation stent use (b = 0.17), preoperative ICA diameter (b = −0.11), and preoperative ECA diameter (b = 0.23). No prior studies have documented preoperative and postoperative ECA diameters in angiography. De Brost et al. [11] reported >50% ECA stenosis in Duplex Doppler Ultrasound, increasing from 29.9% at 3 months to 65.8% at 60 months post-CAS, with greater progression in overstented versus contralateral ECA. ECA stenosis also occurred more frequently in patients with in-stent ICA restenosis [11]. Similarly, Willfort-Ehringer et al. [15] observed > 70% ECA stenosis, increasing from 19.3% to 38.3% at 3 and 24 months post-CAS, respectively. Moreover, previous hemodynamic studies have shown that implanted stents may significantly reduce blood perfusion to the ECA while rapidly restoring ICA flow. After stenting, the ECA demonstrated more extensive regions of disturbed hemodynamics, including retrograde flow, low wall shear stress, a high oscillatory shear index, and elevated relative residence time, compared with the ICA [15]. These alterations were suggested to predispose the ECA to vascular injury and occlusion after CAS [11]. Furthermore, such findings emphasize that factors such as bifurcation geometry and vessel angles may influence ECA patency following intervention. Our identified predictive factors, including preoperative ECA and ICA diameters, suggest that a wider ECA lumen may facilitate plaque displacement from the ICA, increasing narrowing risk. Additionally, second-generation stents with an additional mesh layer may cause hemodynamic disturbances, further contributing to ECA narrowing.

Although de novo ECA occlusion post-CAS occurred in only 2.5% of our patients—consistent with the 0.6–3.8% range reported in prior studies [9,11,16]—it remains a clinically relevant complication. Given the ECA’s role in collateral cerebral perfusion, even rare occlusions may impact select patients, particularly in cases of ICA restenosis. Importantly, we identified preoperative ECA diameter as a negative predictor of occlusion (OR = 0.24), with a cut-off value of 3.05 mm (AUC = 93%) on ROC analysis. This finding supports the role of anatomical assessment in pre-CAS planning. Moreover, studies have reported higher severe stenosis and occlusion rates after CAS (4–28%) compared to CEA (0.4–11%) [9,17], underscoring the need for routine ECA monitoring, especially in patients with borderline vessel diameters.

Given the ECA’s role in collateral cerebral perfusion, its anatomical characteristics, preoperative diameter, and flow dynamics may directly influence outcomes following CAS. In our cohort, ECA occlusion was associated with higher rates of stroke (8.9% vs. 2.1%), TIA (8.7% vs. 1.8%), and ISR (17.4% vs. 2.5%). Although prior studies did not demonstrate significant differences, this may reflect limited sample sizes—Brown et al. [16] reported eight ECA occlusions (3.8%) among 210 CAS patients, and de Brost et al. [11] observed only two cases (0.6%) in 312 patients. Notably, Reid et al. [18] described resolution of recurrent neurological symptoms after ECA stenting in three patients with ICA occlusion. Similarly, Xu et al. [14] and Dong et al. [19] reported improvements in neurological and cognitive status following ECA revascularization in cases of coexisting ipsilateral ICA occlusion. Therefore, as suggested by experts’ opinions [20] and the results of our study, the careful preoperative assessment of ECA morphology, diameter, and flow dynamics may be essential in selecting the most appropriate therapeutic approach (CAS vs. CEA, stent type, or the need for adjunctive ECA intervention), ultimately contributing to better neurological outcomes.

Considering the findings of our study, the observed association between ECA diameter and post-CAS outcomes suggests that current strategies for ICA revascularization may benefit from refinement. ECA diameter could serve as an additional anatomical parameter when evaluating patients for CAS or CEA. In our cohort, an ECA diameter below 3.05 mm was associated with higher rates of ECA occlusion and adverse neurological outcomes. Although these findings require confirmation in prospective, multicenter trials, they indicate that in selected patients with reduced ECA diameter, CEA may warrant consideration over CAS. Until further evidence becomes available, ECA assessment may serve as a helpful adjunct in preprocedural planning to support individualized treatment decisions.

Despite its relatively large cohort of 963 patients, this study has several limitations. Its retrospective, non-randomized, single-center design introduces potential selection bias and limits the generalizability of our findings. The lack of a control group prevents direct comparison with CEA or alternative treatment approaches. Moreover, the observational nature of the study limits control over confounding variables and may lead to incomplete data capture. These factors should be considered when interpreting the results. Prospective, multicenter randomized trials are warranted to validate the role of ECA diameter in guiding ICA revascularization strategies.

## 5. Conclusions

This large, retrospective single-center study of 963 patients demonstrates that CAS leads to significant morphological alterations in the ECA, including frequent overstenting, a consistent reduction in vessel diameter postoperatively, and the occurrence of de novo occlusions. These de novo ECA occlusions were associated with a higher incidence of adverse clinical events, including stroke, TIA, and ISR, during follow-up. Importantly, preoperative ECA diameter was identified as an independent predictive factor for both postoperative ECA narrowing and occlusion. The identification of a diagnostic threshold for ECA diameter further supports its relevance as a measurable risk indicator in the context of CAS. Thus, preprocedural assessment of ECA diameter—particularly values below 3.05 mm—should be considered a key element of treatment planning, potentially favoring CEA over CAS in selected patients.

## Figures and Tables

**Table 1:** Patients’ general characteristics.

Variable	*n* (%), Median (Range), IQR
Age (years)	70 (45–93), IQR 9
Gender	
Male	623 (64.7%)
Female	340 (35.3%)
Current cigarette smoking	392 (40.7%)
Presence of comorbidities (yes)	940 (97.6%)
Arterial hypertension	899 (93.4%)
Dyslipidemia	833 (86.5%)
Diabetes mellitus	305 (31.7%)
Symptomatic ICA stenosis	372 (38.6%)
Stroke	300 (31.2%)
TIA	85 (8.8%)
Other symptoms	
Dizziness	192 (19.9%)
Headache	86 (8.9%)
Syncope	64 (6.7%)
Tinnitus	25 (2.56%)

Abbreviations: ICA—internal carotid artery; IQR—interquartile range; TIA—transient ischemic attack.

**Table 2:** Periprocedural characteristics and postoperative outcomes.

Variable	*n* (%), Mean/Median (Range) SD/IQR
Preoperative ICA diameter (mm)	2.8 (0.4–9.2), IQR 1.6
Postoperative ICA diameter (mm)	6.6 (2.7–11) IQR 1.7
Difference in post- and preoperative ICA diameters (mm)	3.6 (0–8.6), IQR 1.9
Side of the procedure	
Left ICA	518 (53.8%)
Right ICA	445 (46.2%)
Stent used	
1st generation	404 (41.9%)
2nd generation	559 (58.1%)
Stent length	
60 mm	1 (0.1%)
50 mm	1 (0.1%)
40 mm	652 (67.7%)
30 mm	309 (32.1%)
Stent width	
6 mm	2 (0.2%)
7 mm	285 (29.6%)
8 mm	435 (45.2%)
9 mm	177 (18.4%)
10 mm	64 (6.7%)
Covering of ECA orifice by a carotid stent	
Yes	928 (96.4%)
No	35 (3.6%)
ECA diameter before CAS (mm)	4.7 (0.7–12.9), IQR 2
ECA diameter after CAS (mm)	3.8 (0–9.5), IQR 2.4
Difference between pre- and postoperative ECA diameters (mm)	0.8 (−2.7–6.7), IQR 1.7
ECA fate after CAS (*n* = 928)	
Decrease in the diameter	666 (71.7%)
ECA occlusion	23 (2.5%)
No change in the diameter	200 (21.6%)
Increase in the diameter	62 (6.7%)
Patients’ clinical outcome	
Follow-up time (months)	22.57 (0.03–80.2) SD 21.16
Ipsilateral Stroke	22 (2.3%)
30-day stroke	9 (0.9%)
Follow-up stroke	13 (1.4%)
TIA	19 (2%)
30-day TIA	14 (1.5%)
Follow-up TIA	5 (0.5%)
MI	29 (3%)
30-day MI	1 (0.1%)
Follow-up MI	28 (2.9%)
In-stent restenosis	26 (2.7%)
Mortality	14 (1.5%)
30-day mortality	7 (0.7%)
Follow-up mortality	7 (0.7%)

Abbreviations: CAS—carotid artery stenting; ECA—external carotid artery; ICA—internal carotid artery; IQR—interquartile range; MI—myocardial infraction SD—standard deviation; TIA—transient ischemic attack.

**Table 3:** Correlations between ICA and ECA diameters.

Correlations	R	*p*
Preoperative ICA/ECA diameter	0.1	
Preoperative ECA diameter/postoperative ICA diameter	0.12	<0.001
Preoperative ICA diameter/postoperative ECA diameter	0.13	<0.001
Postoperative ICA/ECA diameter	0.17	<0.001
Preoperative ICA diameter/difference between post and -preoperative ECA diameter	−0.07	0.04
Postoperative ICA diameter/difference between post and -preoperative ECA diameter	−0.07	0.03
Preoperative ECA diameter/difference between post and -preoperative ECA diameter	0.22	<0.001
Postoperative ECA diameter/difference between post and -preoperative ECA diameter	−0.44	<0.001

Abbreviations: ECA—external carotid artery; ICA—internal carotid artery.

**Table 4:** Uni- and multivariate logistic regression analysis of predictive factors for postoperative changes in ECA diameter.

	Univariate Analysis				Multivariate Analysis		
Variable	R^2^	b	SE	*p*	b	SE	*p*
Predilatation	0.004	0.19	0.09	0.04	0.1	0.09	0.26
2nd-generation stents	0.006	0.19	0.08	0.02	0.17	0.08	0.02
Stent width	0.002	0.05	0.05	0.24			
Stent length	0.001	−0.01	0.01	0.29			
Preoperative ICA diameter	0.005	−0.07	0.03	0.03	−0.10	0.03	0.001
Preoperative ECA diameter	0.097	0.22	0.02	<0.001	0.23	0.023	<0.001

**Abbreviations**: ECA—external carotid artery; ICA—internal carotid artery; SE—standard error.

**Table 5:** Univariate logistic regression analysis of the predictive factors for ECA occlusion.

	Univariate Analysis		
Variable	OR	95% CI	*p*
Predilatation			0.38
Yes	1.50	0.61–3.7	
No	1		
Stent generation			0.51
1st generation	1.34	0.56–3.18	
2nd generation	1		
Stent width	0.56	0.32–0.98	0.04
Stent length	1.03	0.94–1.12	0.579
Preoperative ICA diameter	0.99	0.69–1.40	0.93
Preoperative ECA diameter	0.24	0.16–0.36	<0.001

**Abbreviations**: CI—Confidence Interval; ECA—external carotid artery; ICA—internal carotid artery; OR—Odds ratio.

**Table 6:** Comparison of clinical outcomes between patients with de novo occlusion and without.

Variable	ECA Occlusion (n = 23)	No ECA Occlusion (n = 940)	*p*
Ipsilateral stroke	2 (8.7%)	20 (2.1%)	0.04
TIA	2 (8.7%)	17 (1.8%)	0.01
MI	1 (4.4%)	27 (2.9%)	0.68
In-stent restenosis	4 (17.4%)	23 (2.5%)	<0.001
Death	1 (4.4%)	13 (1.4%)	0.24

Abbreviations: ECA—external carotid artery; MI—myocardial infraction; TIA—transient ischemic attack.

## Data Availability

The data are available from the corresponding author on request.

## Supplementary Materials

The following supporting information can be downloaded at: https://www.mdpi.com/article/10.3390/jcm14186682/s1, Supplementary Materials S1. Protocol for measurement of ECA and ICA diameters.
