# Supplementary material for: Clinical Impact of External Carotid Artery Remodeling Following Carotid Artery Stenting
Source: J Clin Med. 2025 Sep 22;14(18):6682. doi: 10.3390/jcm14186682 (PMC12470279; doi:10.3390/jcm14186682)
Supplement: Supplementary file 1 [file jcm-14-06682-s001.zip › jcm-3865124-supplementary.pdf]

Supplementary Materials S1. Protocol for Measurement of ECA and ICA Diameters

Measurement Sites and Technique

Diameters of the external carotid artery (ECA) and internal carotid artery (ICA) were measured based on standardized digital subtraction angiography (DSA) images obtained during the index carotid artery stenting (CAS) procedure. All measurements were performed using calibrated digital calipers in a DICOM viewer system with submillimeter resolution.

- **ECA Measurement:**
  The diameter of the ECA was measured at a fixed anatomical point —in the narrowest point of the ECA proximal to the origin of the first visible branch, the superior thyroid artery. This location was selected to ensure consistency across patients and time points (pre- and post-procedural imaging).
- **ICA Measurement:**
  The ICA diameter was measured at the site of maximal luminal narrowing, as determined by visual inspection of orthogonal angiographic projections. The most stenotic segment was identified and selected for measurement.

Repetition and Averaging

Each arterial segment was measured **three times** in the same imaging sequence, with the **arithmetic mean** of the three values recorded as the final measurement for both pre- and post-stenting evaluations.

Blinding Procedure

All measurements were performed by a single investigator who was **blinded to all clinical and procedural data**, including patient symptoms, indication for treatment, and outcomes. The observer was provided with anonymized angiographic image sets, which were encoded with study identifiers only.
